# Dietary rosemary extract modulated gut microbiota and influenced the growth, meat quality, serum biochemistry, antioxidant, and immune capacities of broilers

**DOI:** 10.3389/fmicb.2022.1024682

**Published:** 2022-10-19

**Authors:** Yang Liu, Chuang Li, Xuan Huang, Xu Zhang, Ping Deng, Guitao Jiang, Qiuzhong Dai

**Affiliations:** Hunan Institute of Animal Husbandry and Veterinary Medicine, Changsha, China

**Keywords:** rosemary extract, broilers, growth performance, meat quality, serum biochemistry, antioxidant, immune, gut microbiota

## Abstract

After the legislative ban on the utilization of antibiotics in animal feed, phytochemical substances gained increasing attention as alternatives to antibiotics because of their bioactivities and safety for animals. The present study aimed to investigate the influence of dietary rosemary extract (RE) on growth performance, meat quality, serum biochemistry, antioxidant and immune capacities, and gut microbiota composition of broilers. By exploring connections among RE, physiological characteristics of broilers, and key microbiota, we sought to provide evidence for the utilization of RE in poultry feed. A total of 280 1-d-old female AA broilers were randomly separated into five groups, and were fed a basal diet supplemented with 0, 250, 500, 750, and 1,000 mg/kg of RE, respectively. Results showed that with regard to growth performance, both 500 and 750 mg/kg RE reduced the broiler feed-to-gain ratio from 1 to 21 d (*P* = 0.018). Regarding meat quality, all compositions of dietary RE reduced cooking loss of breast muscle (*P* < 0.01), and 500 and 1,000 mg/kg RE reduced the cooking loss of thigh muscle (*P* = 0.045). Regarding serum biochemical indexes, 500 mg/kg RE reduced ALB, TCHO, HDL-C, and LDL-C, and 750 mg/kg RE reduced GLU, TP, ALB, UA, TG, TCHO, HDL-C, and LDL-C (*P* < 0.01). Regarding antioxidant and immune capacities, 250, 500, 750, and 1,000 mg/kg RE increased T-AOC, GSH-Px, SOD, CAT, IL-2, IgA, IgG, and IgM levels (*P* < 0.01), and decreased serum MDA level (*P* < 0.01). RE at 750 mg/kg showed similar effects on growth performance, meat quality, and antioxidative and immune capacities, but a better influence on serum biochemical indexes of broilers compared with 500 mg/kg. Further analysis was conducted to investigate the effect of 750 mg/kg dietary RE on the gut microbial composition of broilers, and the results showed that 750 mg/kg RE reduced the relative abundance of *g_Lachnoclostridium*, *g_Escherichia_Shigella*, and *g_Marvinbryantia* (*P* <0.05, LDA score >2), which were negatively correlated to antioxidative and immune-associated parameters (*P* < 0.05). In conclusion, 750 mg/kg dietary RE was shown to have certain beneficial effects on growth performance and meat quality, and hypolipidemic and hypoglycemic effects on broilers. Furthermore, dietary RE improved antioxidant and immune capacities, which was partially attributed to the reduced abundance of certain pathogenic bacteria in broilers.

## Introduction

In recent years, modern intensive farming systems had gradually replaced traditional free-ranging and backyard farming systems, which satisfied the increasing demands for poultry products in the world market (Robins and Phillips, 2011). However, it also affected the health of birds, leading to physiological stress, digestive dysfunction, and intestinal microbiome disorder (Averós and Estevez, 2018; Wang S. et al., 2018), which led to low animal product quality and incurred losses to the breeders. Recent studies found that negative conditions, such as hydrogen sulfide and ammonia, brought by intensive production system in chicken houses also reduced the antioxidant and immune capacities of chickens (Zheng et al., 2019; Han et al., 2020; Shah et al., 2020; Wang et al., 2022; Zhang et al., 2022). For past decades, antibiotic additives were commonly used in feed to attenuate stress and promote the growth of birds (Castanon, 2007), but continuous misuse of antibiotics led to serious food safety issues. So, many countries banned the utilization of antibiotics in poultry feed (Witte, 2000; Hayes et al., 2004). Therefore, it is now an urge to develop new feed additives to replace antibiotics in the poultry industry.

Rosemary (*Rosmarinus officinalis* L.) is a medicinal plant belonging to the *Lamiaceae* family. The phytochemicals in rosemary extract (RE) are mainly phenolic compounds, di- and triterpenes, including rosmarinic acid, camphor, caffeic acid, ursolic acid, carnosic acid, and carnosol (Ulbricht et al., 2010). Previous literature stated that RE exerted various pharmacological activities, such as antibacterial (Bozin et al., 2007), antidiabetic (Bakrel et al., 2008), ant-inflammatory (Amaglo et al., 2010), antitumor (Tai et al., 2012), and antioxidant effects (Pérez-Fons et al., 2010). In the past years, RE was widely used in medication and food preservation because rosemary was cheap, easily available, and non-toxic (Oluwatuyi et al., 2004). Also, RE showed great potential as food and animal feed additives, since it could decrease lipid peroxidation and oxidative stress in animals, thus demonstrating a strong antioxidative effect (Yildirim et al., 2018; Yang et al., 2021). However, the influence of dietary RE at variable concentrations on growth performance and intestinal microbiome homeostasis of animals was not consistent (Turan and Yiğitarslan, 2016; Yildirim et al., 2018). Therefore, the present study was designed to study the influence of dietary RE at different levels on growth performance, meat quality, nutrient digestibility, antioxidative and immunological capacities, and intestinal microbiota construction of broilers, and to provide evidence for the use of RE as a broiler feed additive.

## Materials and methods

The animal experiment of this study was approved by the Hunan Institute of Animal Husbandry and Veterinary Medicine (Changsha, Hunan, China) under protocol code AHVM20210801.

### Experimental design and diets

A total of 280 female AA broilers at 1 d of age were selected for the present experiment, and were kept in plastic plain netting cages (1.8 m × 1.2 m × 2 m) with free access to water and feed. Birds were randomly separated into five groups. Each group had seven cages, which were treated as seven replicates, with eight broilers in each cage. The experiment lasted 42 d, in which 1–21 d was the early stage, and 22–42 d was the late stage. The treatments included a control group (CON) which was fed the basal diet supplemented with no RE, and four treatment groups (including RE250, RE500, RE750, and RE1000) which were fed a basal diet supplemented with 250, 500, 750, and 1,000 mg/kg RE, respectively. The compositions and nutrient levels of the basal diets for two stages of broiler growth were based on the nutritional requirements for broilers (National Research and Council [NRC], 1994) with some modifications, which are given in Table 1. The RE used in the study was kindly provided by Hunan Zhizhiyuan Agriculture and Animal Husbandry Co., LTD. (Luxi, Hunan, China). It is a liquid-form complex extracted from rosemary leaves and flowers, whose main active ingredients are 2.5% ursolic acid and 1.5% oleanolic acid. The diets were pelletized after all ingredients were completely mixed. The maintenance of the experimental facility was conducted according to the standard procedures of a commercial broiler farm. Briefly, the temperature was set at 33°C for the first week and dropped 2-3°C each week until the temperature reached 20-22°C, which was maintained for the rest of the experimental period. Ventilation was open every day to keep the moisture and humidity of the facility at a suitable level.

**TABLE 1:** The composition and nutrient levels of diets (air-dry basis, %).

Items	Diets	
	Early stage (1–21d)	Late stage (22–42 d)
**Ingredients**		
Corn	49.13	46.4
Flour	12.0	15.0
Soybean meal	27.0	22.0
Extruded soybean	5.0	5.0
Corn gluten meal (CP 60%)	2.0	3.0
Soybean oil	0	4.0
Calcium bicarbonate	1.5	1.5
Calcium phosphate	1.3	1.1
NaCl	0.3	0.3
70% L-Lysine H2SO4	0.82	0.78
98% DL-Methionine	0.23	0.22
98% L-Threonine	0.21	0.19
Phytase	0.01	0.01
Premix^1^	0.5	0.5
Total	100	100
**Nutrient levels^2^**		
ME (Mcal/kg)	2.93	3.21
Crude protein (%)	20.63	19.18
Ca (%)	1.01	0.95
Total protein (%)	0.60	0.55
Available phosphorus (%)	0.36	0.31
Lysine (%)	1.40	1.25
Methionine (%)	0.51	0.48
Threonine (%)	0.91	0.82

^1^Provided per kg of diet: VA 15,000 IU, VD_3_ 7,000 IU, VE 80 mg, VK_3_ 12.5 mg, VB_1_ 1.87 mg, VB_2_ 6.25 mg, VB_6_ 5 mg, VB_12_ 0.025 mg, niacin 25 mg, calcium pantothenic acid 25 mg, folic acid 0.62 mg, biotin 0.4 mg, VC 120 mg, choline 500 mg, Fe 80 mg, Cu 15 mg, Zn 90 mg, Mn 90 mg, I 0.25 mg, Se 0.25 mg, and Co 0.1 mg.

^2^ME was calculated values, and the other nutrient levels were measured values.

### Growth performance

The broilers were weighed based on the cage on 1, 21, and 42 d after being fasted for 12 h. The amount of feed intake for each cage was measured and recorded every day. The average daily weight gain (ADG), average daily feed intake (ADFI), and feed-to-gain ratio (F/G) for broilers at the early stage, late stage, and whole period were calculated by cages to represent the growth performance of broilers.

### Meat quality

On the 42 d, one broiler from each cage (seven broilers for each group) was randomly selected for sampling. The broilers were slaughtered by cervical dislocation after blood collection. Chunks of 2-cm-thick right-side leg muscle and breast muscle were cut from each broiler for meat quality analysis. Meat color was determined using a colorimeter (CR-400, Minolta Camera Co., Osaka, Japan) as L*, a*, and b* after 45-min postmortem, which were indicators of lightness, redness, and yellowness, respectively. The water-holding capacity, cooking loss, and shear force of meat samples at 45-min postmortem were assessed according to the method described previously (Qiao et al., 2017). Briefly, the water-holding capacity was represented by the drip loss, which was measured based on the weight loss of the meat sample after being squeezed in a determinator (MAEC-18, Nanjing Mingao Instrument Co., Nanjing, China) setting at 35 kg force for 5 min. The cooking loss was measured by the weight loss of the meat sample after being cooked in an 80°C water bath. The shear force was measured by using a texture analyzer (C-LM4-1, Beijing Bulader Tech Co., Beijing, China) through shearing perpendicular to the myofibers of the cooked meat sample.

### Serum biochemical, immunological, and antioxidative parameters

Five milliliters of blood samples was collected from the wing veins of each sampled broiler (seven birds per treatment) and were kept in separate tubes. The blood samples were kept at room temperature for 1 h and then centrifuged at 4,000 × *g* for 5 min to obtain the serum samples. Commercial assay kits (Hangzhou Adicon Medical Laboratory Center Co., LTD., Hangzhou, China) and an automatic biochemical analyzer (URIT-8000, Hangzhou Adicon Medical Laboratory Center Co., LTD., Hangzhou, China) were used to measure the serum levels of biochemical parameters, including glucose (GLU), total protein (TP), albumin (ALB), uric acid (UA), triglyceride (TG), total cholesterol (TCHO), high-density lipoprotein cholesterol (HDL-C), low-density lipoprotein cholesterol (LDL-C), and free fatty acid (FFA).

Commercial assay kits (Nanjing Jiancheng Bioengineering Institute, Nanjing, China) and an automated fluorescence instrument (Multiskan*™* Skyhigh, Thermo Fisher Scientific, Waltham, USA) were used to determine the serum levels of immunological parameters, including interleukin-2 (IL-2), immunoglobulin A (IgA), immunoglobulin G (IgG), and immunoglobulin M (IgM), as well as oxidative stress-related parameters, including glutathione peroxidase (GSH-Px), superoxide dismutase (SOD), catalase (CAT), malondialdehyde (MDA), and total antioxidant capacity (T-AOC).

### Gut microbiota composition

After slaughtering, the digesta in the cecum of each broiler sample in CON and RE750 (seven replicates in each treatment) was collected separately into 2 mL tubes, frozen immediately in liquid nitrogen, and shipped in dry ice to Shanghai Bioprofile Biotechnology Co., Ltd. (Shanghai, China) for 16S rRNA gene sequencing. Briefly, the microbial DNA was extracted from digesta samples with E.Z.N.A. Bacterial DNA Kit (Omega Bio-tech, Norcross, GA, USA) according to the manufacturer’s instructions. The primer pairs 338F (5′-ACTCCTACGGGAGGCAGCAG-3′) and 806R (5′-GGACTACHVGGGTWTCTAAT-3′) were designed to amplify the V3 – V4 regions of the bacterial 16S rRNA gene, and then the purified amplicons were paired-end sequenced on an Illumina MiSeq PE300 platform (Illumina, San Diego, CA, USA). QIIME (version 1.17) software was used to filter the obtained sequences from the samples, and the high-quality sequences were clustered into operational taxonomic units (OTUs) with a cutoff of 97% similarity using UPARSE (version 7.1). The alpha diversities of the cecal microbiota were estimated at the genus level. Linear discriminant analysis effect size (LEfSe) was performed to identify the differences in bacterial taxa between groups. Spearman correlation analysis was applied to evaluate the relationship of microbial communities with the measured parameters.

### Statistical analysis

A cage was used as the experimental unit. Statistical analysis of data was done with Statistical Package for the Social Sciences (SPSS) 19.0 (IBM, Armonk, USA). One-way ANOVA followed by Duncan’s multiple range test was performed to test the significant mean differences among groups. Polynomial orthogonal contrasts were applied to determine the linear and quadratic responses of measured parameters to the dietary RE concentration. All results were presented as mean values and pooled standard errors of the mean values (SEM). A probability of *P* < 0.05 was considered significant.

## Results

### Growth performance

The growth performance-associated parameters are presented in Table 2. In the early stage, the body weight (*P* = 0.060) and ADG (*P* = 0.058) of broilers in the RE500 and RE750 groups showed an increasing trend when compared to the animals in the CON, RE250, and RE1000 groups. Moreover, F/G in RE500 and RE750 was significantly lower than that in CON and RE1000 (*P* = 0.018), and a significant quadratic relationship was noticed between the F/G and dietary RE levels (*P* = 0.004). Growth performance parameters of broilers in the late stage or during the whole period showed no differences among treatments (*P* > 0.050).

**TABLE 2:** Effects of rosemary extract on the growth performance of broilers^1^.

Item	Treatment					SEM	*P*-value		
	CON	RE250	RE500	RE750	RE1000		Treatment	Linear	Quadratic
**Body weight (g)**									
1 d	47.37	47.72	48.16	48.23	48.58	0.17	0.159	0.009	0.032
21 d	798.49	792.09	825.61	811.79	787.14	4.66	0.060	0.912	0.098
42 d	2573.99	2535.92	2539.44	2594.91	2536.43	0.01	0.514	0.886	0.986
**Early stage**									
ADG (g)	35.79	35.45	37.02	36.36	35.17	0.23	0.058	0.826	0.101
ADFI (g)	50.22	48.99	48.66	49.38	50.36	0.35	0.584	0.754	0.179
F/G	1.34^ab^	1.32^abc^	1.25^c^	1.29^c^	1.36^a^	0.01	0.018	0.687	0.004
**Late stage**									
ADG (g)	84.55	83.04	81.61	84.91	83.30	0.66	0.416	0.918	0.717
ADFI (g)	133.36	130.33	128.90	130.45	134.57	1.04	0.487	0.699	0.129
F/G	1.59	1.57	1.59	1.54	1.62	0.01	0.547	0.787	0.588
**Whole period**									
ADG (g)	60.17	59.24	59.32	60.63	59.23	0.36	0.507	0.868	0.983
ADFI (g)	91.79	89.66	88.22	89.92	92.47	0.73	0.423	0.718	0.107
F/G	1.53	1.51	1.49	1.48	1.56	0.01	0.240	0.641	0.072

ADG, average daily weight gain; ADFI, average daily feed intake; F/G, feed-to-gain ratio.

^1^Results were based on seven samples per treatment.

^a–c^Mean values within a row bearing different superscripts differ significantly (*P* < 0.05).

### Meat quality

The meat quality-associated parameters under the influence of dietary RE are presented in Table 3. All RE-treated broilers showed significantly lower cooking losses in the breast muscle compared to the CON (*P* < 0.001) group, and significant linear (*P* < 0.001) and quadratic (*P* < 0.001) relationships were noticed between breast muscle cooking loss and dietary RE level. In addition, the thigh muscle cooking losses in RE500 and RE1000 were significantly lower when compared to CON (*P* = 0.045), and also significant linear (*P* = 0.017) and quadratic (*P* = 0.038) relationships were noticed between thigh muscle cooking loss and dietary RE level. Other meat quality-associated parameters were not different among the groups (*P* > 0.05).

**TABLE 3:** Effects of rosemary extract on the meat quality of broilers^1^.

Item		Treatment					SEM	*P*-value		
		CON	RE250	RE500	RE750	RE1000		Treatment	Linear	Quadratic
**Breast muscle**										
Water holding capacity (%)		31.93	32.98	36.86	37.29	39.31	1.18	0.118	0.353	0.651
Cooking loss (%)		26.06^a^	21.48^b^	19.37^b^	19.69^b^	17.59^b^	0.91	< 0.001	<0.001	< 0.001
Shear force (N)		52.01	49.02	48.76	42.00	44.93	1.80	0.318	0.324	0.573
Meat color	L*	48.18	47.78	47.40	48.15	49.03	0.40	0.715	0.625	0.453
	a*	5.62	6.05	6.56	5.92	5.55	0.25	0.666	0.850	0.398
	b*	6.42	6.42	6.63	6.49	6.85	0.18	0.941	0.610	0.822
**Thigh muscle**										
Water holding capacity (%)		33.59	32.61	37.07	38.98	40.65	1.64	0.540	0.250	0.450
Cooking loss (%)		25.46^a^	21.62^ab^	19.78^b^	22.39^ab^	19.11^b^	0.73	0.045	0.017	0.038
Shear force (N)		38.39	28.22	34.64	35.68	37.02	1.40	0.090	0.572	0.226
Meat color	L*	54.17	55.47	54.75	55.58	54.30	0.45	0.826	0.807	0.491
	a*	8.68	7.99	9.05	8.52	8.49	0.33	0.995	0.999	0.996
	b*	8.26	6.94	8.01	8.40	8.06	0.24	0.507	0.637	0.590

^1^Results were based on seven samples per treatment.

^a,b^Mean values within a row bearing different superscripts differ significantly (*P* < 0.05).

L*, a*, b* represent the brightness, redness, and yellowness of meat.

### Serum biochemical parameters

The effect of dietary RE on the serum biochemical parameters is presented in Table 4. Significant quadratic relationships were noticed between dietary RE levels and serum levels of ALB, TCHO, HDL-C, and LDL-C (*P* < 0.05), and a significant linear relationship was noticed between dietary RE levels and serum levels of UA (*P* = 0.048). Specifically, serum levels of GLU and TP in RE750 were significantly lower than those in CON, RE250, and RE1000 (*P* < 0.01). Serum levels of ALB, TCHO, HDL-C, and LDL-C in RE500 and RE750 were significantly lower than those in CON, RE250, and RE1000 (*P* < 0.01). Serum levels of UA and TG in RE750 were significantly lower than those in other groups (*P* < 0.01).

**TABLE 4:** Effects of rosemary extract on serum biochemical indexes of broilers^1^.

Item	Treatment					SEM	*P*-value		
	CON	RE250	RE500	RE750	RE1000		Treatment	Linear	Quadratic
GLU (mmol/L)	7.73^bc^	9.50^ab^	6.26^cd^	4.73^d^	9.72^a^	0.42	<0.01	0.794	0.064
TP (g/L)	25.41^ab^	29.01^a^	20.53^bc^	19.07^c^	29.64^a^	1.08	<0.01	0.850	0.063
ALB (g/L)	9.39^b^	10.76^ab^	6.89^c^	5.70^c^	11.89^a^	0.51	<0.01	0.988	0.011
UA (μmol/L)	293.29^ab^	338.14^a^	257.57^b^	185.14^c^	279.00^ab^	13.08	<0.01	0.048	0.088
TG (mmol/L)	0.61^b^	0.80^a^	0.59^b^	0.42^c^	0.73^ab^	0.03	<0.01	0.531	0.498
TCHO (mmol/L)	3.69^b^	3.68^b^	2.74^c^	2.49^c^	4.49^a^	0.15	<0.01	0.711	<0.001
HDL-C (mmol/L)	1.64^b^	1.67^ab^	1.10^c^	0.95^c^	2.10^a^	0.09	<0.01	0.754	<0.001
LDL-C (mmol/L)	1.31^b^	1.44^ab^	0.79^c^	0.80^c^	1.75^a^	0.08	<0.01	0.680	0.001
FFA (mmol/L)	339.03	403.99	346.80	302.52	426.65	16.98	0.124	0.547	0.554

GLU, glucose; TP, total protein; ALB, albumin; UA, uric acid; TG, triglyceride; TCHO, total cholesterol; HDL-C, high-density lipoprotein cholesterol; LDL-C, low-density lipoprotein cholesterol; FFA, free fatty acid.

^1^Results were based on seven samples per treatment.

^a–c^Mean values within a row bearing different superscripts differ significantly (*P* < 0.05).

### Serum oxidative stress-related parameters

The effects of dietary RE on serum oxidative stress-related parameters are presented in Table 5. Compared to the CON group, all RE-treated groups demonstrated significantly higher levels of T-AOC, GSH-Px, SOD, and CAT, and a significantly lower level of MDA in serum (*P* < 0.01). In addition, significant linear and quadratic relationships were found between dietary RE levels and the serum antioxidative parameters (*P* < 0.05). Regarding the comparisons among the RE-treated groups, the serum level of T-AOC in RE500 was significantly higher than that in RE250 (*P* < 0.01). The serum level of GSH-Px in RE500 was significantly higher than that in RE250 and RE1000 (*P* < 0.01). The serum level of CAT in RE500 was significantly higher than that in RE1000 (*P* < 0.01). Serum level of MDA in RE500 was significantly lower than that in RE1000 (*P* < 0.01).

**TABLE 5:** Effects of rosemary extract on serum antioxidative parameters of broilers^1^.

Item	Treatment					SEM	*P*-value		
	CON	RE250	RE500	RE750	RE1000		Treatment	Linear	Quadratic
T-AOC (mmol/L)	0.47^c^	0.58^b^	0.65^a^	0.63^ab^	0.60^ab^	0.01	<0.01	0.001	<0.001
GSH-Px (ng/ml)	32.03^c^	43.20^b^	49.72^a^	46.18^ab^	42.98^b^	1.27	<0.01	0.004	<0.001
SOD (ng/ml)	1.85^b^	2.41^a^	2.53^a^	2.51^a^	2.35^a^	0.05	<0.01	0.001	<0.001
CAT (pg/ml)	122.27^c^	173.16^ab^	190.73^a^	178.11^ab^	167.71^b^	5.03	<0.01	0.005	<0.001
MDA (nmol/L)	3.04^a^	2.34^bc^	2.08^c^	2.20^bc^	2.49^b^	0.07	<0.01	0.014	<0.001

T-AOC, total antioxidant capacity; GSH-Px, glutathione peroxidase; SOD, superoxide dismutase; CAT, catalase; MDA, malondialdehyde.

^1^Results were based on seven samples per treatment.

^a–c^Mean values within a row bearing different superscripts differ significantly (*P* < 0.05).

### Serum immunological parameters

The effects of dietary RE on the serum immunological parameters are presented in Table 6. Similar to the antioxidative parameters, all RE-treated groups showed significantly higher levels of immunological parameters in serum compared to those in CON (*P* < 0.01). Moreover, both linear and quadratic relationships between dietary RE level and the serum immunological parameters were found to be significant (*P* < 0.01). Among the RE-treated groups, the serum level of IgM in RE500 was significantly higher than that in RE250. The serum level of IgG in RE500 was significantly higher than that in RE250 and RE1000 (*P* < 0.01). Serum levels of IgA and liver levels of IL-2 in RE500 and RE750 were significantly higher than those in RE250 and RE1000 (*P* < 0.01).

**TABLE 6:** Effects of rosemary extract on serum immunological parameters of broilers^1^.

Item	Treatment					SEM	*P*-value		
	CON	RE250	RE500	RE750	RE1000		Treatment	Linear	Quadratic
IL-2 (pg/ml)	65.00^b^	87.25^a^	90.37^a^	92.64^a^	84.69^a^	2.06	<0.01	0.001	<0.001
IgM (μg/ml)	120.42^c^	169.33^b^	190.28^a^	184.39^ab^	173.82^ab^	4.91	<0.01	<0.001	<0.001
IgG (μg/ml)	447.48^c^	559.48^b^	656.96^a^	615.80^ab^	567.51^b^	14.67	<0.01	0.003	<0.001
IgA (μg/ml)	56.09^c^	69.76^b^	79.08^a^	78.65^a^	67.93^b^	1.84	<0.01	0.01	<0.001

IL-2, interleukin-2; IgM, immunoglobulin M; IgG, immunoglobulin G; IgA, immunoglobulin A.

^1^Results were based on seven samples per treatment.

^a–c^Mean values within a row bearing different superscripts differ significantly (*P* < 0.05).

### Gut microbiota composition

**FIGURE 1:**
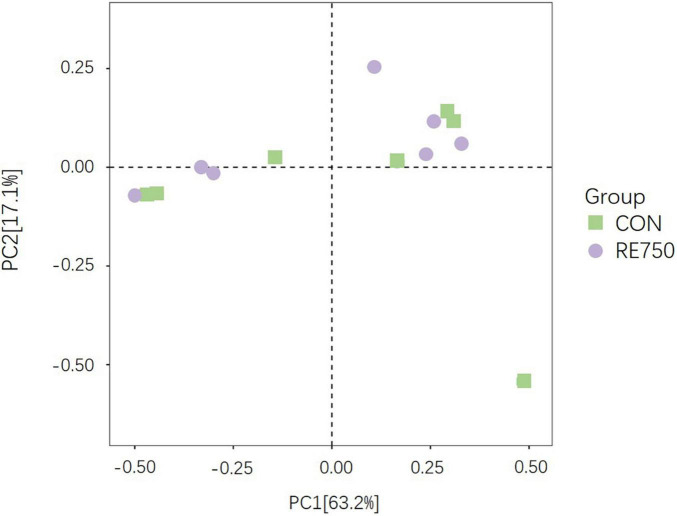
Principal component analysis ordinated plots of gut microbiota in broilers of control group and RE750.

Based on the effects of dietary RE on growth performance, meat quality, serum biochemical parameters, antioxidative, and immunological parameters of broilers, it was concluded that dietary supplementation of RE at 750 mg/kg demonstrated advanced benefits on broilers. Therefore, we further investigated the difference in gut microbiota composition between broilers in CON and RE750. The α diversity indexes were not significantly different (*P* > 0.05), as shown in Table 7, and the β diversity presented by principal component analysis (PCA) revealed no clear clustering patterns for gut microbiota between CON and RE750 (Figure 1), which suggested that the microbial diversity of broilers between CON and RE750 was similar.

**TABLE 7:** The α diversity index of gut microbiota in broilers of CON and RE750^1^.

Index	Treatments		SEM	*P*-value
	CON	RE750		
Shannon	6.99	6.32	0.50	0.526
Simpson	0.89	0.87	0.03	0.667
Chao	1717.19	1874.07	125.54	0.554

^1^Results were based on seven samples per treatment.

**FIGURE 2:**
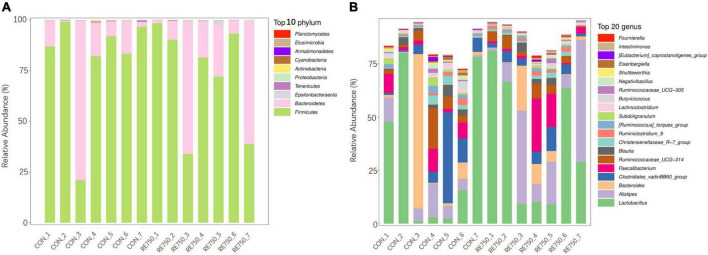
Gut microbiota composition of broilers in control group and RE750. The top 10 most abundant bacteria at the phylum **(A)** and top 20 most abundant bacteria at the genus levels **(B)**.

The composition of gut microbiota in broilers of CON and RE750 at phyla and genera levels is shown in Figure 2. The most predominant bacterial phylum in both groups was *p_Firmicutes*, which accounted for 80.13 and 72.60% microbial population in broilers of CON and RE750, respectively. The second most abundant phylum was *p_Bacteroidetes*, which constituted 19.09 and 26.60% of the microbial population in broilers of CON and RE750, respectively. Regarding the microbiota composition at the genera level, the top five most predominant genera were *g_Lactobacillus* (32.57% in CON and 38.37% in RE750), *g_Alistipes* (6.60% in CON and 20.86% in RE750), *g_Bacteroides* (12.08% in CON and 5.44% in RE750), *g_Clostridiales_vadinBB60_group* (12.28% in CON and 4.78% in RE750), and *g_Faecalibacterium* (4.59% in CON and 6.80% in RE750).

**FIGURE 3:**
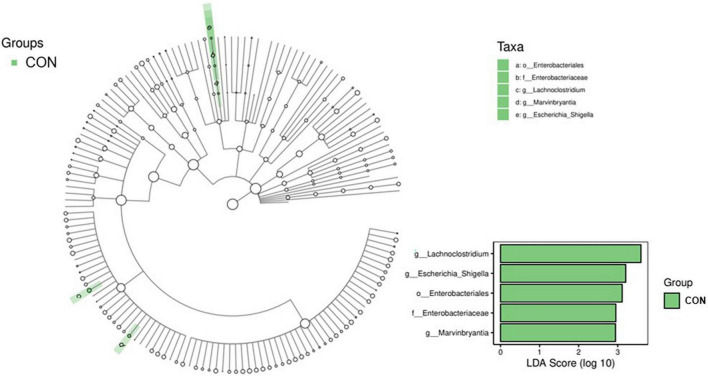
Significantly changed bacteria between broilers in control group and RE750 revealed by LEfSe analysis. Only a linear discriminant analysis (LDA) score of >2 was considered significant.

**FIGURE 4:**
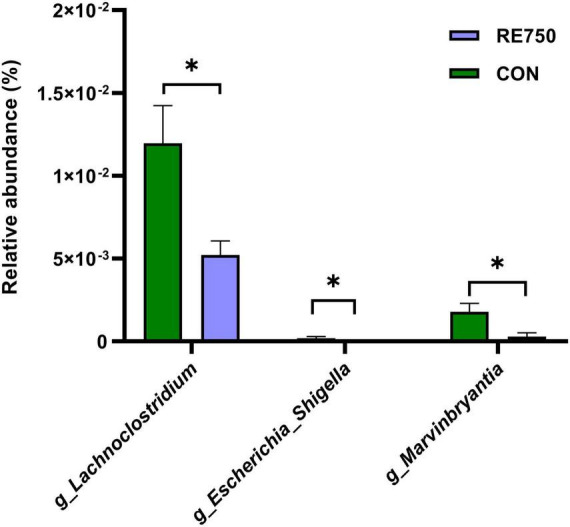
The relative abundance of *g_Lachnoclostridium*, *g_Escherichia_Shigella*, and *g_Marvinbryantia* in control group and RE750. Data were subjected to *t*-test analysis, and results are shown as mean ± SEM (*n* = 7). Significant correlation was marked by *P≤0.05.

A LEfSe analysis was conducted to determine the potential biomarker in different groups, and the results are shown in Figure 3. Three bacterial genera, including *g_Lachnoclostridium* (belonging to *f_Enterobacteriaceae* and *o_Enterobacteriales*), *g_Escherichia_Shigella*, and *g_Marvinbryantia*, were potential biomarkers for CON (*P* <0.05, LDA score >2). The comparison of the relative abundance of *g_Lachnoclostridium*, *g_Escherichia_Shigella*, and *g_Marvinbryantia* in CON and RE750 is shown in Figure 4, which indicated that 750 mg/kg RE significantly reduced the relative abundance of these bacteria.

### Correlation analysis between gut microbiota and measured parameters

**FIGURE 5:**
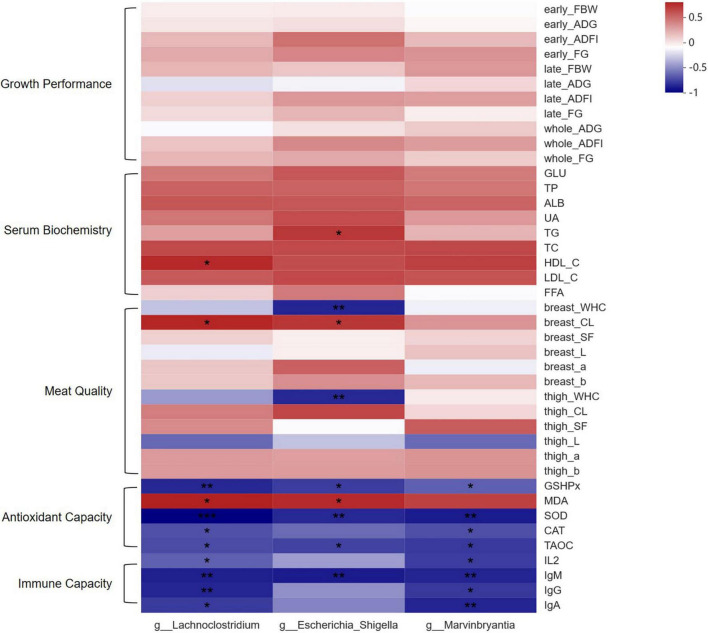
Spearman correlation heatmap of significantly different microbiota between CON and RE750 and the measured parameters. The intensity of the colors demonstrated the degree of association (blue: negative correlation, red: positive correlation). Significant correlations were marked by * (0.01 < P ≤ 0.05), ** (0.001 < P ≤ 0.01), or *** (P < 0.001). FBW, final body weight; ADG, average daily weight gain; ADFI, average daily feed intake; FG, feed-to-gain ratio; GLU, glucose; TP, total protein; ALB, albumin; UA, uric acid; TG, triglyceride; TCHO, total cholesterol; HDL-C, high-density lipoprotein cholesterol; LDL-C, low-density lipoprotein cholesterol; FFA, free fatty acid; WHC, water holding capacity; CL, cooking loss; SF, shearing force; GSH-Px, glutathione peroxidase; MDA, malondialdehyde; SOD, superoxide dismutase; CAT, catalase; T-AOC, total antioxidant capacity; IL-2, interleukin-2; IgM, immunoglobulin M; IgG, immunoglobulin G; IgA, immunoglobulin A.

Spearman correlation analysis was conducted to determine the relationships between the biomarker and measured parameters related to growth performance, serum biochemistry, meat quality, antioxidant capacity, and immune capacity of the broilers, which is shown in Figure 5. The majority of antioxidative and immune-related parameters were negatively correlated to the number of three differential bacteria (*P* <0.05). Serum MDA level was positively associated with the number of three differential bacteria (*P* <0.05). In addition, the cooking loss of breast muscle was positively associated with the number of *g_Lachnoclostridium* and *g_Escherichia_Shigella* (*P* <0.05), and water holding capacities of breast and thigh muscles were negatively associated with the number of *g_Escherichia_Shigella* (*P* <0.05). Moreover, HDL-C level in serum was positively associated with the number of *g_Lachnoclostridium* (*P* <0.05), and TG level in serum was positively associated with the number of *g_Escherichia_Shigella* (*P* <0.05).

## Discussion

The present study investigated the effects of different levels of dietary RE on growth performance, meat quality, serum biochemistry, antioxidative and immunological capacities, and gut microbiota composition of broilers. The results showed that dietary RE at 250, 500, 750, and 1,000 mg/kg had no negative effects on the growth performance of broilers at the late stage (22–42 d) and whole stage (1-42 d) (*P* >0.05). However, dietary RE at 500 and 750 mg/kg reduced significantly the F/G ratio of broilers at an early stage (1–21 d) when compared to the animals treated with no dietary RE (*P* = 0.018), indicating that dietary RE at an appropriate concentration (500 and 750 mg/kg), rather than high level (1,000 mg/kg), had a promoting effect on the growth of broilers at an early stage. The regression analysis also supported our finding by showing a significant quadratic relationship between the dietary RE level and F/G ratio of broilers at an early stage (*P* = 0.004). This result was consistent with a previous study that RE supplemented in drinking water at a low rate (1.5 mL/L), but not high rates (3 or 6 mL/L), reduced the feed conversion rate of broilers during 1–21 d compared to the animals drinking natural water or water with sodium nitrate (Akhavast and Daneshyar, 2017). Several studies also confirmed the promotional effects of dietary RE on the growth performance of New Zealand White rabbits and weaning pigs, which might be explained by improved nutrient digestibility, oxidant and immune capacities, intestinal morphology, and gut microbiota composition of the treated animals (Yang et al., 2021; Elazab et al., 2022).

Dietary RE showed no deteriorated effects on the meat quality of broilers (*P*>0.05). However, 250, 500, 750, and 1,000 mg/kg RE significantly decreased the cooking loss of breast muscle (*P* <0.001), while 500 and 1,000 mg/kg RE significantly decreased the cooking loss of leg muscle (*P* = 0.045). In addition, dietary RE levels had significant linear and quadratic relationships with the cooking losses of both breast and leg muscles (*P* <0.05). Cooking loss measurement represented the most rapid and important method to estimate meat juiciness after cooking (Barbera and Tassone, 2006). Results in the present study indicated that the dietary RE could improve the meat quality of broiler meat, especially juiciness. A similar result was obtained by Yesilbag et al. (2011) that dietary supplementation with rosemary and its volatile oil could improve broiler meat quality. An earlier study stated that 350 ppm of RE maintained low values of TBARS and total carbonyl content, and delayed aldehyde formation in chicken meat patties due to its antioxidative effect (Al-Hijazeen and Al-Rawashdeh, 2017). Also, another study on lamb concluded that rosemary distillation residues could increase vitamin E content after intake, which enhanced the oxidative status and improved the fatty acid profile of the meat (Yagoubi et al., 2021).

Serum biochemistry analysis further explored that 750 mg/kg dietary RE significantly reduced the serum GLU, TG, TCHO, HDL-C, and LDL-C levels in broilers (*P* <0.01). Meanwhile, quadratic relationships were found among serum TCHO, HDL-C, LDL-C levels, and dietary RE levels (*P* < 0.05). The findings indicated that proper levels of dietary RE might have hypoglycemic and hypolipidemic activities on broilers, as reported in earlier literature (Dearlove et al., 2008). GLU is the main energy source for the organism, and TG is the main energy-storing substance in the organism. RE could trigger insulin secretion to stimulate GLU utilization by peripheral tissues and GLU uptake by cells, consequently leading to a reduction in serum GLU level (Emam, 2012). A study on human hepatocyte HepG2 cell reported that RE could suppress DGAT1 activity and intracellular TG synthesis, the two major enzymes responsible for TG synthesis (Cui et al., 2012), which could be a possible explanation for the hypolipidemic effect of RE. Torki et al. (2018) reported that rosemary oil reduced the serum concentration of TG in broilers under thermoneutral conditions. Another study found that serum GLU and TCHO levels decreased in broilers fed rosemary oil, which could possibly be explained by the fact that the body started using energy from lipolysis, as decreased GLU levels could not meet the physiological needs (Belenli et al., 2015). Manafi et al. (2014) also reported that rosemary oil supplementation in diets showed good effects on reducing TCHO, HDL, and LDL levels in broiler chickens. However, another study reported that rosemary powder supplementation decreased the TG, TCHO, and LDL-C levels, but increased HDL-C levels in the blood of 52-week-old laying hens (Alagawany and Abd El-Hack, 2015). The inconsistent results in HDL-C levels under the influence of RE might be attributed to the different experimental animals or the forms of RE used in the studies. In addition, the present study found that dietary RE level had a quadratic relationship with the serum ALB level (*P* – 0.011), and a quadratic trend with the serum TP level (*P* = 0.063). Dietary RE at 250 and 1,000 mg/kg increased, but 500 and 750 mg/kg decreased, the serum TP and ALB levels (*P* <0.01). A previous study also found that 0.5% rosemary leaves meal supplemented in the diet significantly increased the TP in the blood of broilers, but as the level of rosemary leaves increased, the blood TP level gradually decreased (Ghazalah and Ali, 2008). These results suggested that a low level of dietary RE might have the effect of increasing the protein reservation in broilers. Moreover, 750 mg/kg of dietary RE significantly reduced the serum UA level in broilers (*P* <0.01), which was in line with previous studies (Ghazalah and Ali, 2008; El-Boshy et al., 2012). Assessment of this renal biomarker showed that proper levels of dietary RE might have certain renoprotective effects as well (Naimi et al., 2017).

Besides biochemical parameters, antioxidative and immunological cytokines were also important indicators reflecting the health status of birds. The immunological and oxidative stresses were inevitable in birds raised in intensive farming systems, which disturbed the redox balance, triggered the innate and adaptive immune responses, and eventually took up excessive nutrition and energy from growth (Kuo et al., 2011; Chen et al., 2019; Lauridsen, 2019; Liu et al., 2021). Within the organism, an antioxidant defense system regulates the redox balance, which consisted of enzymatic components, such as SOD, CAT, and GSH-Px, and non-enzymatic components, such as GSH (Surai et al., 2019). The redox balance in poultry is responsible for the regulation of various physiological and biochemical processes, including cell signaling, gene expression, and homeostasis maintenance (Corsello et al., 2018; Miao et al., 2022). Once the ROS production exceeds the ability of the antioxidant defense system to neutralize them, oxidative stress occurs and important biological molecules, including polyunsaturated fatty acids, proteins, and DNA can be damaged, leading to detrimental consequences in terms of health, growth, and development of animal (Surai et al., 2019; Cui et al., 2022). MDA was formed by ROS degrading the polyunsaturated lipids in the organism, which was considered as a common biomarker of oxidative stress in animals (Jing et al., 2013). Lymphocyte cells in the avian innate immune system were able to produce a diversity of antibodies, such as IgM, IgG, and IgA, to serve as the first line of defense to fight against inflammation (Berghof et al., 2018). In the adaptive immune system, type-1 helper T cells (Th1) bind to the macrophages and release IL-2, which activates the bound T cells to eliminate inflammation (Jacob and Pescatore, 2017). In the present study, dietary RE quadratically increased the levels of antioxidant-related parameters in the serum of broilers, including T-AOC, GSH-Px, SOD, and CAT (*P* <0.01), and decreased the serum MDA level (*P* <0.01), suggesting that dietary RE could improve the antioxidative capacity of broilers. Also, dietary RE quadratically increased the levels of immune function-related parameters in the serum of broilers. Previous studies had proved the antioxidative effects of RE in pigs (Liotta et al., 2015), quails (Cetin et al., 2017), food preservation (Sebranek et al., 2005), and immune-modulating effects of RE in rats (Silva et al., 2014) and *in vitro* model (Yi and Wetzstein, 2010). The active components of RE mainly contributed to its antioxidative effect, including classes of phenolic acids, flavonoids, diterpenoids, and so on (Nieto et al., 2018). The isoprenoid quinones in the components of RE act as chain terminators of free radicals and chelators of ROS (Wu et al., 1982), and the phenolic compound can turn lipid and hydroxyl radicals into stable products (Gordon, 1990). More studies regarding the antioxidative effects of specific active components of RE are available, such as ursolic acid (Nascimento et al., 2013), rosmarinic acid (Muñoz-Muñoz et al., 2013), carnosic acid (Doolaege et al., 2011), oleanolic acid (Wang et al., 2020), and so on.

The gut microbial homeostasis was required for the development and maturation of the animals, which affected a wide range of physiology aspects, including growth performance, production performance, nutrient digestibility, immune function, and antioxidative capacity (Sommer and Bäckhed, 2013; Fathima et al., 2022). Previous literature reported that RE had the potential to reshape the gut microflora in mice under stressed conditions (Guo et al., 2018; He et al., 2022), and rosemary leaf meal could increase the population of *Lactobacillus* but reduce the population of *E. coli* and *Salmonella* (Ogwuegbu et al., 2021). However, in the present study, 750 mg/kg dietary RE did not change the α or β diversity of the gut microflora in broilers (*P* >0.05). The inconsistent results might be attributed to the differences in species and the health conditions of the experimental animals, as well as the forms of rosemary supplementation in the diet. Even so, it was observed that 750 mg/kg dietary RE created shifts in gut microbial composition, such as the relative abundance of *p_Firmicutes*, *p_Bacteroidetes*, and associated genera. With the LEfSe analysis, the relative abundance of *g_Lachnoclostridium*, *g_Escherichia_Shigella*, and *g_Marvinbryantia* was significantly reduced by 750 mg/kg dietary RE (*P* <0.05, LDA score >2), which suggested a hypothesis that the three genera in the broilergut might be potential biomarkers under the effects of dietary RE. Correlation analysis disclosed that the three significantly differential bacteria genera were negatively associated with antioxidant and immune capacity related-parameters. Similar results were reported that the relative abundance of *g_Lachnoclostridium* was higher in pig gut under heat stress (Xia et al., 2022) and in mice with ulcerative colitis carcinogenesis (Wang et al., 2019). A recent study reported that an increased level of *g_Lachnoclostridium* significantly correlated to increased frequencies of T helper (Th) 1 and Th2 cells (Beukema et al., 2022), whose excessive responses could lead to the development of inflammatory or autoimmune diseases (Miao et al., 2021; Sun et al., 2022). It indicated that *g_Lachnoclostridium* had immune-stimulatory effects and might be responsible for the Th1- and Th2-inducing effects. The possible mechanism could be that *g_Lachnoclostridium-*derived antigens or metabolic products enhance Th1 and Th2 responses (Berer et al., 2018). A previous study stated that *g_Marvinbryantia* was associated with inflammatory cell infiltration in rat colon (Wang et al., 2020). It was also found that the relative abundance of *g_Marvinbryantia* was positively correlated with the levels of MDA in serum and inflammatory factors in the intestinal mucosa, and negatively correlated with the antioxidant capacities in serum and the concentrations of organic acids in the intestinal contents of pigs fed with antibiotics (Wang Y. et al., 2018). Previous research on broilers found that *g_Escherichia_Shigella* induced lung tissue inflammation by activating NLRP3 inflammasome and promoting IL-1β release (Liu et al., 2020). Besides, *g_Escherichia_Shigella* impaired intestinal structure and induced various pro-inflammatory pathways, such as the secretion of virulence factors, resulting in infection and diarrhea in the host (Gong et al., 2019). Our research showed reductions in the relative abundance of *g_Lachnoclostridium*, *g_Escherichia_Shigella*, and *g_Marvinbryantia* under the influence of 750 mg/kg dietary RE, which could possibly contribute to the enhancement of immune and antioxidative capacities in broilers.

## Conclusion

In conclusion, the present study suggested that RE supplemented in the diet showed no negative effects on growth performance and meat quality of broilers, but rather decreased the F:G ratio at the early stage and the cooking losses of both breast and thigh muscles. Dietary RE also quadratically decreased the energy metabolism and lipid metabolism of the broilers, showing hypolipidemic and hypoglycemic effects. One possible explanation for the beneficial effects of dietary RE on broilers could be it improved the antioxidant capacity and immune functions, which could be partially attributed to the fact that dietary RE reduced the relative abundance of *g_Lachnoclostridium*, *g_Escherichia_Shigella*, and *g_Marvinbryantia*. However, further studies were required to approve the functions of these bacteria to verify the interactions among dietary RE, gut microbiota, and the antioxidative and immunological capacities of the host.

## Data availability statement

The datasets presented in this study can be found in online repositories. The names of the repository/repositories and accession number(s) can be found in the article/supplementary material.

## Ethics statement

The animal study was reviewed and approved by Hunan Institute of Animal Husbandry and Veterinary Medicine (protocol code: AHVM20210801).

## Author contributions

GJ and QD: conceptualization, supervision, and funding acquisition. GJ: methodology. QD: validation, and project administration. YL: formal analysis, and original draft preparation. CL: investigation. XH: resources. XZ: data curation. YL, GJ, and QD: reviewing and editing. PD: visualization. All authors have read and agreed to the published version of the manuscript.
